# Exploring the landscape of *Babesia bovis* vaccines: progress, challenges, and opportunities

**DOI:** 10.1186/s13071-023-05885-z

**Published:** 2023-08-10

**Authors:** John Harvey M. Santos, Hannah V. Siddle, Ali Raza, Danielle I. Stanisic, Michael F. Good, Ala E. Tabor

**Affiliations:** 1 The University of Queensland, Queensland Alliance for Agriculture & Food Innovation, Centre for Animal Science, St Lucia, Qld 4072 Australia; 2 Griffith University, Institute for Glycomics, Southport, Qld 4215 Australia; 3 The University of Queensland, School of Chemistry & Molecular Biosciences, St Lucia, Qld 4072 Australia

**Keywords:** Bovine babesiosis, *Babesia bovis*, Vaccine development, Immune response

## Abstract

Bovine babesiosis, caused by different *Babesia* spp. such as *B. bovis*, *B. bigemina*, *B. divergens*, and *B. major*, is a global disease that poses a serious threat to livestock production. *Babesia bovis* infections are associated with severe disease and increased mortality in adult cattle, making it the most virulent agent of bovine babesiosis. *Babesia bovis* parasites undergo asexual reproduction within bovine red blood cells, followed by sexual reproduction within their tick vectors, which transmit the parasite transovarially. Current control methods, including therapeutic drugs (i.e., imidocarb) have been found to lead to drug resistance. Moreover, changing environmental factors add complexity to efficient parasite control. Understanding the fundamental biology, host immune responses, and host–parasite interactions of *Babesia* parasites is critical for developing next-generation vaccines to control acute disease and parasite transmission. This systematic review analyzed available research papers on vaccine development and the associated immune responses to *B. bovis*. We compiled and consolidated the reported vaccine strategies, considering the study design and rationale of each study, to provide a systematic review of knowledge and insights for further research. Thirteen studies published since 2014 (inclusive) represented various vaccine strategies developed against *B. bovis* such as subunit, live attenuated, and viral vector vaccines. Such strategies incorporated *B. bovis* proteins or whole live parasites with the latter providing the most effective prophylaxis against bovine babesiosis. Incorporating novel research approaches, such as "omics" will enhance our understanding of parasite vulnerabilities.

**Graphical Abstract:**

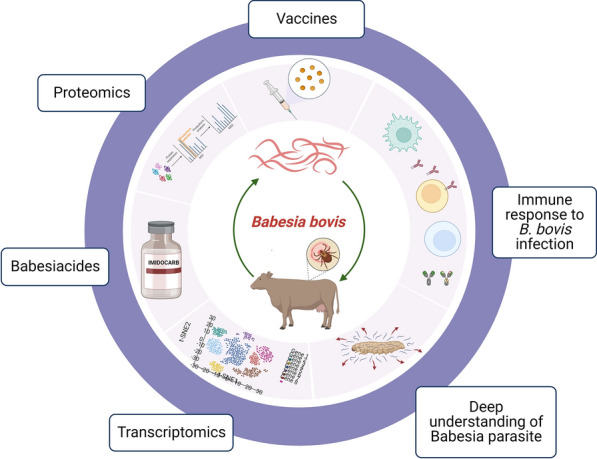

**Supplementary Information:**

The online version contains supplementary material available at 10.1186/s13071-023-05885-z.

## Introduction

*Babesia bovis* is one of the most significant causative agents of babesiosis in cattle, with severe impacts on livestock industries. Substantial economic losses range from US$ 22 million in Australia to US$ 60 million in China [1]. *Babesia bovis* infection can cause significant economic losses due to its high morbidity and mortality rates in cattle. Because of its high prevalence in countries like China, Brazil, Turkey, and Thailand, poor identification and diagnosis of the parasites leads to cattle losses. The disease is transmitted by the tick *Rhipicephalus microplus* species complex, prevalent in tropical and subtropical regions in America, Africa, Australia, and Asia, where it is the most important ectoparasite of cattle [2]. Calves possess a level of immunity related to innate immune and age-related factors that remains for 6–8 months, while adult cattle are most at risk and are susceptible to clinical disease. Cattle infected with *B. bovis* can exhibit clinical signs which include fever, anemia, hemoglobinuria, lethargy, loss of appetite, and weight loss, leading to decreased milk and meat production [2, 3]. Infection with *B. bovis* can result in a high mortality rate, especially in susceptible breeds or animals not previously exposed to the pathogen [2]. However, certain *Bos indicus* cattle breeds indigenous to the *Babesia* endemic regions often possess a certain degree of natural resistance to the disease, thus resulting in mild to moderate disease clinical signs [2].

**Fig. 1:**
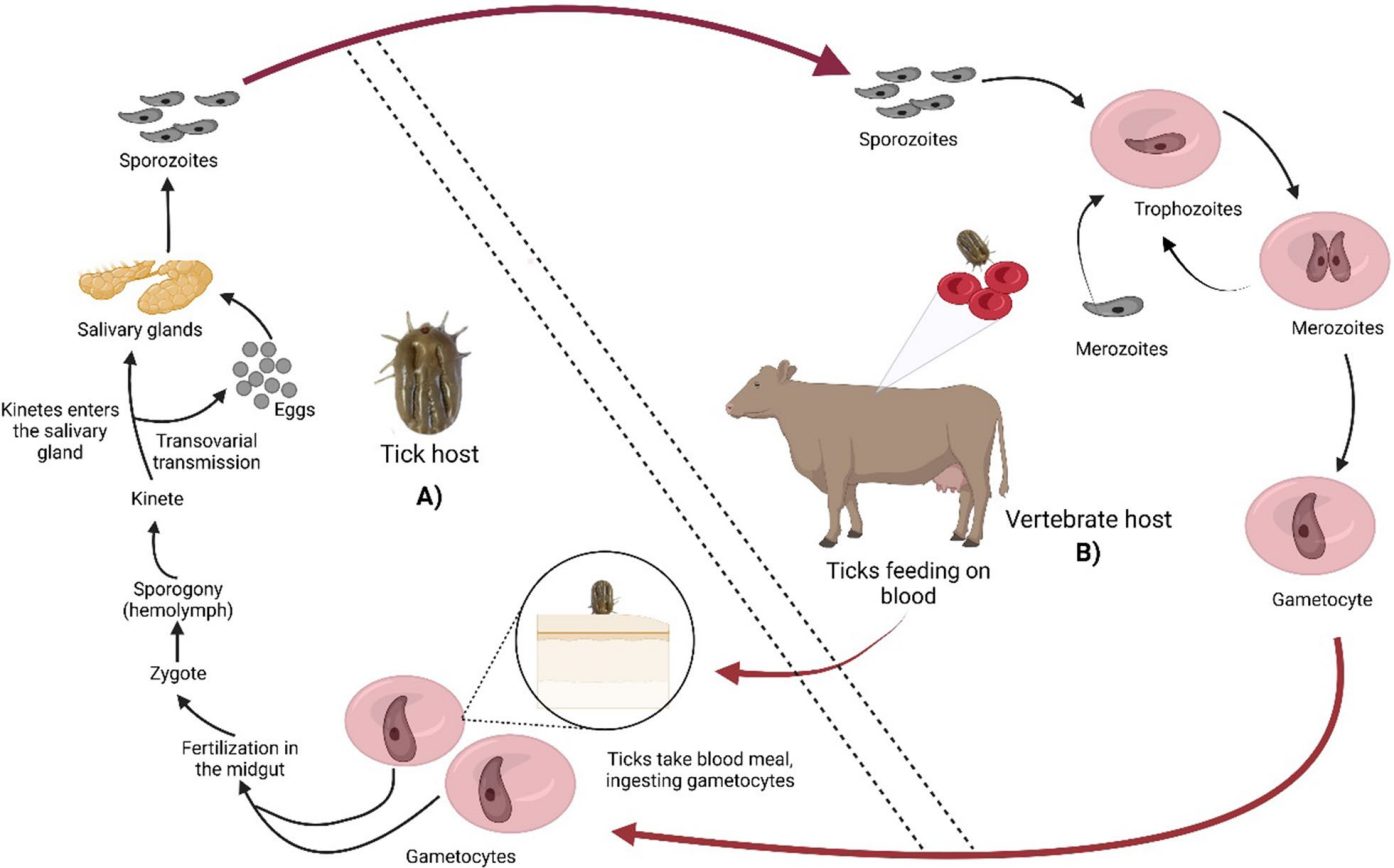
Life cycle of *Babesia bovis*. *Babesia bovis* has two main phases: **A** The sexual cycle takes place in the tick host. The sexual cycle of *B. bovis* is initiated when a tick ingests gametocytes of the parasite during its blood meal on a mammalian host. Following ingestion, the gametocytes differentiate into male and female gametes within the tick's gut, which fuse to form a zygote. The zygote develops into an ookinete that migrates to the tick's salivary glands as a kinete, where it may be transmitted to a new vertebrate host during tick feeding. Kinetes gain access to the hemolymph of the tick, replicate, and invade various organs. Additionally, transovarial transmission of *B. bovis* can also occur within the tick. Subsequently, the kinetes develop into invasive sporozoites, which are transmitted by the tick to a new vertebrate host during a blood meal. **B** The asexual cycle occurs in the mammalian (vertebrate) host. During the asexual cycle of *B. bovis*, the sporozoites invade the red blood cells (RBCs) of the bovine host and develop into trophozoites and divide by binary fission, resulting in the formation of merozoites. These merozoites continue to proliferate, developing into trophozoites and eventually give rise to new merozoites. Within the RBCs, certain merozoites undergo differentiation into male and female gametocytes, which remain within the RBCs of the bovine host. These gametocytes are then acquired by ticks during their feeding process

The life cycle of *B. bovis* infection is complex, involving sexual and asexual phases. The sexual reproductive phase occurs in the tick vector, whereas the asexual reproductive phase happens in the mammalian host [4, 5] (Fig. 1), specifically in the red blood cells (RBCs) [4]. When the zygote develops into an ookinete that migrates to the tick’s salivary glands as a kinete, then these kinetes develop into invasive sporozoites, which are transmitted by the tick to a new vertebrate host during a blood meal. Furthermore, transovarial transmission of *B. bovis* can also occur within the tick. The infection begins when *Babesia* spp. sporozoites enter the RBCs and multiply through asexual reproduction. When merozoites are released from these RBCs, they can reinfect other RBCs within the bovine host. Some of these parasites transform into male and female gametocytes. Once the tick ingests bovine blood containing these gametocytes, the sexual phase of the infection begins in the tick. The zygote, which is formed in the tick midgut when male and female gametocytes fuse, undergoes sporogony, a process that leads to the production of sporozoites [6].

Effective control measures against *B. bovis* infection include tick control through acaricides, vaccination, and babesicidal drugs [7–10]. However, they all have critical limitations. Acaricides are used to target tick vectors, but there is evidence that ticks are developing resistance [11, 12]. Also, in endemic regions, the use of drugs such as imidocarb is limited as they are expensive and cannot be used to prevent infection; in addition, suboptimal dosing might lead to the emergence of drug-resistant parasites, and could leave residual metabolites [13]. In extensive cattle production systems, the timely administration of babesicidal drugs poses a considerable challenge in the presence of clinical cases. Therefore, the combination of these control methods needs to be handled carefully. Countries such as Australia, South Africa, Argentina, Brazil, and Uruguay have utilized and produced live attenuated vaccines [14]. In Australia, prevention is mainly based on the use of a trivalent live vaccine which contains attenuated *B. bovis* and *Babesia bigemina* Australian strains, and *Anaplasma centrale* originally imported from South Africa is used to protect against *Anaplasma marginale* [15]. However, this has many significant limitations such as its short shelf-life of only 4 days from the production date when stored between 2 and 8 °C, possible reversion of the organisms to their virulent form, and issues in standardizing the dose [15]. Thus, further research is needed to develop new and effective control strategies against this disease. Developing an effective vaccine against *B. bovis* involves an understanding of the parasite’s biology and the immune response to infection. Several potential vaccine candidates have been identified including heat shock proteins [16], surface antigens [17, 18], and apical membrane proteins [19]. These different antigens have been shown to induce immune responses, but none to date have demonstrated protection against live pathogenic *B. bovis* challenge. The host’s immune response to the parasite is also a critical factor in the disease prognosis, with the development of immunity being a vital component in controlling the disease. In this systematic review, available research papers on vaccine development studies and the associated immune response to *B. bovis* were critically analyzed. The reported vaccine strategies were compiled and consolidated considering the study design and rationale of each study to provide a systematic review of knowledge and insights for further investigation.

## Methods

### Research selection and search criteria

**Fig. 2:**
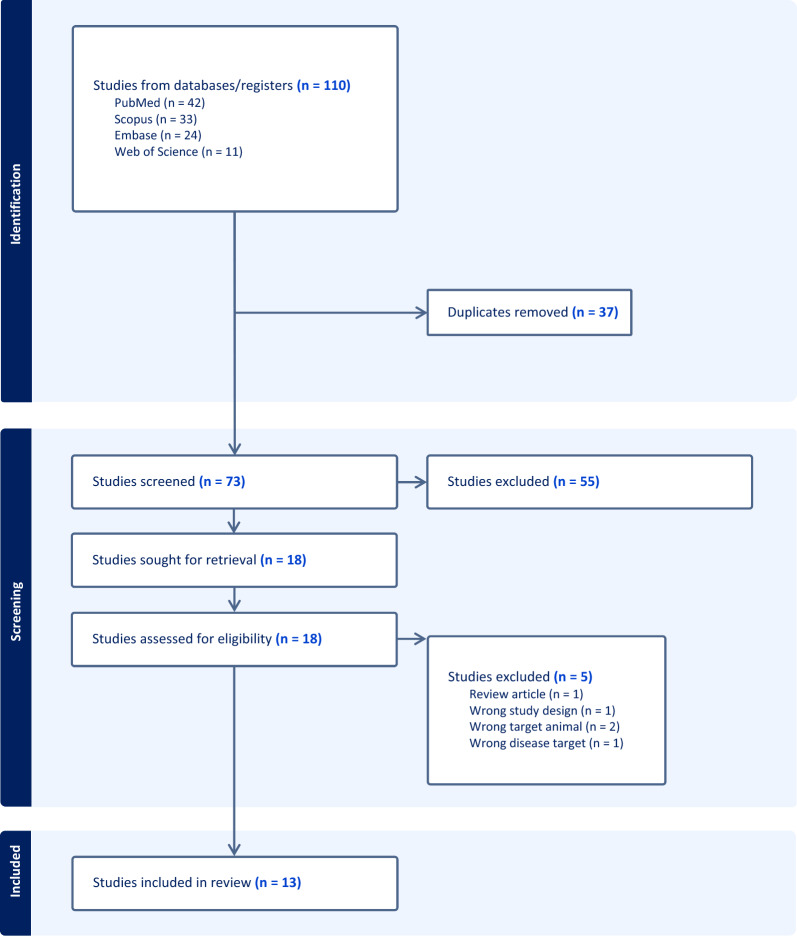
Flow chart of the study selection and identification process. The steps were adopted from the Preferred Reporting Items for Systematic Reviews and Meta-Analyses (PRISMA) guidelines

To collate the available research articles published within the last 10 years reporting *B. bovis* vaccine development and testing in bovines, searches were conducted in four electronic databases (PubMed, Web of Science, Embase, and Scopus) on 23 February 2023. This systematic review was framed around the review title “vaccine candidates for *Babesia bovis*” using the keywords “cattle,” “vaccine,” “babesiosis,” and “*Babesia bovis*” for the searches. The detailed search strategies for each database are listed in Additional file 1. The results generated by the databases were imported into EndNote and the duplicates were removed. In this systematic review, results that did not report original data were excluded, including reviews, conference abstracts, and book chapters. The research articles were downloaded, and articles that did not have English full text available were excluded. The titles and abstracts of all records were screened to filter out studies that performed vaccine tests and longitudinal evaluation and in hosts other than cattle and mice along with antigens derived from pathogens other than *Babesia* spp. The procedure in this systematic review was adopted from the Preferred Reporting Items for Systematic Reviews and Meta-Analyses (PRISMA) (Fig. 2).

### Data extraction

Studies that met our inclusion criteria were subjected to the next phase of screening to extract the following information: journal name, year of publication, and vaccine type.

## Results

### Characteristics of studies included in the review

In total, 110 studies were retrieved from the customized searches. After removing the duplicates, the first screening was performed on 73 studies. Fifty-five studies were removed, including records that did not report original data, records that did not have an English full text, and the inclusion of animals that were not cattle. The full text of the remaining studies (*n* = 18) was further screened for eligibility, and 13 studies were eventually subjected to data extraction. The systematic review identified 13 studies on vaccine development for *B. bovis* that met the inclusion and eligibility criteria.

Table 1 shows the academic journals in which each study was published and the distribution of published research articles across several peer-reviewed journals that focus on the development of a vaccine for *B. bovis*. According to the data, *Parasites & Vectors* and *Vaccine* were the most prolific journals, publishing three papers on the topic, followed closely by *Pathogens* with two publications. The remaining journals, namely *Ticks and Tick-borne Diseases*, *Veterinary Parasitology*, *Frontiers in Immunology*, *Frontiers in Veterinary Science*, and *Revista Brasileira de Parasitologia Veterinária*, had only one publication each. This suggests that researchers have published a modest number of papers on *B. bovis* vaccine development in recent years.

**Table 1:** Number of published papers by journal from 2014 to 2022

Journal name	Number of papers
*Parasites & Vectors*	3
*Vaccine*	3
*Pathogens*	2
*Ticks and Tick-borne Diseases*	1
*Veterinary Parasitology*	1
*Frontiers in Immunology*	1
*Frontiers in Veterinary Science*	1
*Revista Brasileira de Parasitologia Veterinária*	1

**Fig. 3:**
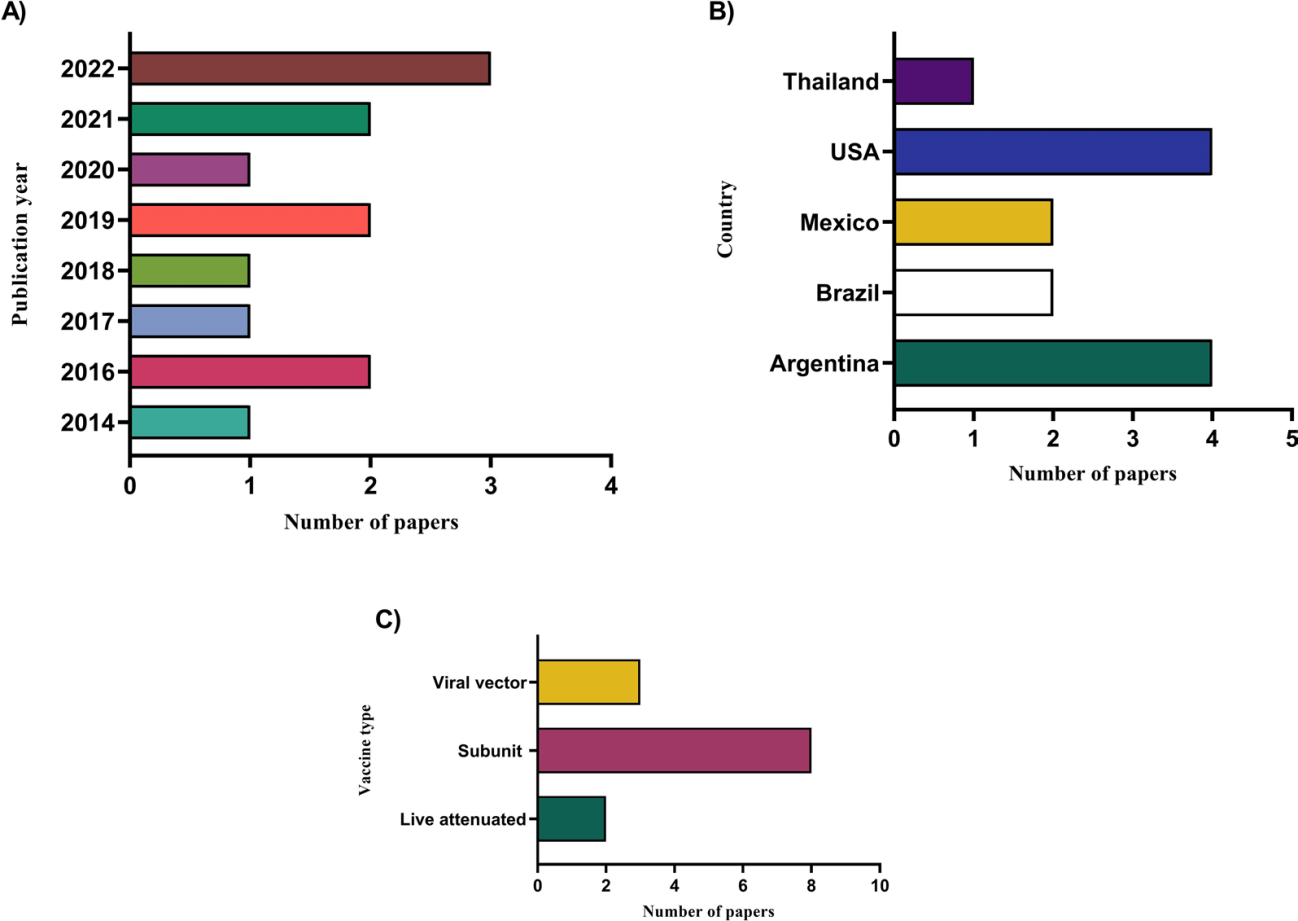
Number of studies categorized into **A** publication year, **B** geographical region, and **C** type of vaccine. The 13 papers were stratified according to their publication year, geographical region, and vaccine type used in the study

The *B. bovis* vaccine development papers included in this systematic review were published between 2014 and 2022. Figure 3A presents the number of papers published, indicating a general upward trend in the number of publications, with a marked increase between 2020 and 2022. Geographically, the studies reviewed were mainly from Argentina (*n* = 4) and the USA (*n* = 4), which together accounted for 62% (8/13) of the papers included in this review (Fig. 3B). Figure 3C depicts the number of papers stratified by vaccine type, indicating that subunit vaccines (*n* = 8) were the most frequently reported vaccine strategy, accounting for 62% of the reviewed papers. In summary, Fig. 3 suggests that research focused on *B. bovis* vaccine development is expanding, with a growing number of publications in recent years. Geographically, the Americas are at the forefront of this research, and recombinant vaccines appear to be the most widely studied vaccine strategy.

### Characterization of *B. bovis* vaccine development studies

Most studies included in our systematic review reported the development of subunit vaccines (Fig. 3C, Table 2) [7, 18–24]. These subunit vaccines consisted of *B. bovis* membrane proteins, oligosaccharides, and merozoite surface antigens. Regarding live attenuated vaccines, *B. bovis* S74-T3Bo was attenuated and used to immunize cattle [25]. Details of the included studies are provided in Tables 2 and 3.

**Table 2:** Vaccine strategies developed against *B. bovis*

Type of vaccine	Antigen(s)/strain(s) used	Findings	References
Live attenuated	*B. bovis-*attenuated S74 T3Bo strain	New alterations in the composition of immune cells in the bloodstream, as well as changes in the expression of cytokines, have been observed in peripheral blood. These changes are linked to the immune response against acute bovine babesiosis and are indicative of a protective effect	[25]
Subunit	*B. bovis* apical membrane antigen 1 (AMA-1), merozoite surface antigen 2c (MSA-2c), and rhoptry-associated protein 1 (RAP-1) proteins	AMA-1, MSA-2c, and RAP-1 contain conserved epitopes that are recognized by B and T cells. These epitopes trigger the production of neutralizing antibodies and promote a durable Th1 immune response	[21]
Subunit	Structural ectodomains I and II of *B. bovis* apical membrane antigen 1 [BbAMA-1(I/II)]	Cattle that have been immunized with BbAMA-1(I/II) exhibit substantially elevated levels of total immunoglobulin (Ig)G antibodies, along with a heightened ratio of IgG2 to IgG1. Furthermore, this immunization has been found to induce a robust Th1 cell response in the vaccinated cattle	[19]
Live attenuated	Stable transfected strain of *B. bovis* expressing an enhanced green fluorescent protein (eGFP) and a chimeric version of Bm86 (*B. bovis*/Bm86/eGFP)	Post-mortem analysis did not reveal any indication of parasites sequestering in the cerebral capillaries, which confirms that the strain has been attenuated. Additionally, this is the first documented case of *B. bovis* that has been genetically modified to express the tick antigen Bm86 on the surface of merozoites, triggering an antibody response against native Bm86	[26]
Subunit	Synthetic ß-(1 → 6)-linked glucosamine oligosaccharides conjugated to tetanus toxoid (5GlcNH_2_-TT)	Experienced acute babesiosis, characterized by the adherence of infected erythrocytes to capillary vessels in the brain. Despite the production of antibodies against this antigen, they were unable to prevent the onset of the disease	[24]
Subunit	*B. bovis* 6-cysteine proteins A and B	Cattle that were immunized produced antibodies against r6cys A and r6cys B, but these antibodies were ineffective in inhibiting the sexual reproduction of the parasite in ticks	[7]
Subunit	*B. bovis* GPI-anchored surface antigen 1 (GASA-1)	When *B. bovis *in vitro cultures were exposed to anti-GASA-1 antibodies, there was a partial yet significant reduction in erythrocyte invasion. This suggests that the protein GASA-1 contains epitopes that are sensitive to neutralization by antibodies	[20]
Viral vector	*B. bovis* merozoite surface antigen 2c (MSA-2c), rhoptry-associated protein 1 (RAP-1), and heat shock protein 20 (HSP20)	The absence of protective effects observed with this recombinant formulation underscores the importance of conducting additional basic and clinical investigations in the bovine model to attain the desired level of effectiveness	[27]
Subunit	*B. bovis* rhoptry neck protein 2 (RON2)	RON2 as a novel *B. bovis* vaccine candidate antigen that contains conserved B-cell epitopes that elicit partially neutralizing antibodies	[22]
Subunit	*B. bovis* merozoite surface antigens (MSA-1, MSA-2b, and MSA-2c)	Calves up to 6 months of age, all the calves developed active immunity against *B. bovis*	[23]
Subunit	*B. bovis* merozoite surface antigens: MSA-2a1, MSA-2b, and MSA-2c	Elicited invasion-inhibitory antibodies and IFN-γ-producing cells	[18]
Viral vector	Chimeric multi-antigen of DNA fragments containing B- and T-cell epitopes of merozoite surface antigen 2c (MSA-2c), rhoptry-associated protein 1 (RAP-1) and heat shock protein 20 (HSP20) genes	Elevated levels of IgG, IFN-γ, CD4^+^ and CD8^+^ T cells were successfully attained	[28]
Viral vector	Immunodominant B- and T-cell epitopes of three *B. bovis* proteins: merozoite surface antigen 2c (MSA-2c), rhoptry-associated protein 1 (RAP-1), and heat shock protein 20 (HSP20)	The viral-vectored approach alone induced significant levels of IFN-γ and resulted in a higher ratio of IgG2a subclass	[29]

**Table 3:** Promising vaccine development studies and their prospects

Vaccine type	Study	Limitation	Future research direction
Live attenuated	[25]	The absence of significant changes in immune cell behavior and cytokine expression patterns in animals infected with Att-S74-T3Bo and then superinfected with Vir-S74-T3Bo suggests that antibodies might play a role in providing protection during reinfection	• In future vaccine trials, it is recommended to prioritize certain approaches such as using live attenuated parasites, genetically modified live parasites, *Babesia* antigens, and novel adjuvants • These methods should focus on activating myeloid cells (monocytes and neutrophils) and CD4^+^ T cells early on. It is also important to assess the balance of pro- and regulatory cytokines (TNF-α, CXCL10, IFN-γ, IL-10, and IL-4) in the peripheral blood as a potential indicator of protection against acute *Babesia* infection
Subunit	[21]	Despite using peptides of *B. bovis* antigens in vaccines that generate neutralizing immunity and involve CD4^+^ T cells, the resulting Th1 immune response was not effective in protecting vaccinated cattle against a virulent strain of *B. bovis*. This failure may be due to using a single antigen in the vaccine, which may not generate a strong enough immune response to block multiple stages of the invasion process	• In this research, the scientists discovered peptides of MSA2c and AMA-1 that could generate neutralizing antibodies and IFN-γ, which is a Th1 cytokine associated with protection • However, it remains to be determined whether a combination of these peptides in a multi-antigen vaccine could enhance the effectiveness of the immune response, in terms of both humoral and cellular responses, or during exposure to a virulent strain
Subunit	[19]	Different *B. bovis* strains that significantly hinder the effectiveness of live vaccines and pose a challenge in creating subunit vaccines	• Considering that BbAMA-1's structural domains I and II can elicit both humoral and cellular immune responses, especially Th1 responses, BbAMA-1 is a promising vaccine candidate for bovine babesiosis • This is significant because strain variations pose challenges to live vaccines and subunit vaccine development. BbAMA-1, being highly conserved, has the potential to overcome strain-related issues by providing protective immunity against various *B. bovis* strains in real-world conditions
Subunit	[7]	It is worth noting that *B. bovis* may not have all the necessary enzymes for complete *N*-linked glycosylation, unlike the *Plasmodium* parasite. As a result, the native 6cys proteins in *B. bovis* are expected to differ in terms of protein folding, surface glycan profile, stability, and other characteristics compared to recombinant 6cys proteins that undergo significant glycosylation when expressed in HEK 293 cells, which is a eukaryotic system	• Peptides derived from *B. bovis* cys proteins that were used to generate rabbit antibodies may provide protective epitopes • As a result, future research efforts will be directed towards further investigating these specific regions of the 6cys A and 6cys B proteins to gain deeper insights into their immunogenicity and potential for protective immunity

### The significance of understanding immune responses against *B. bovis*

**Fig. 4:**
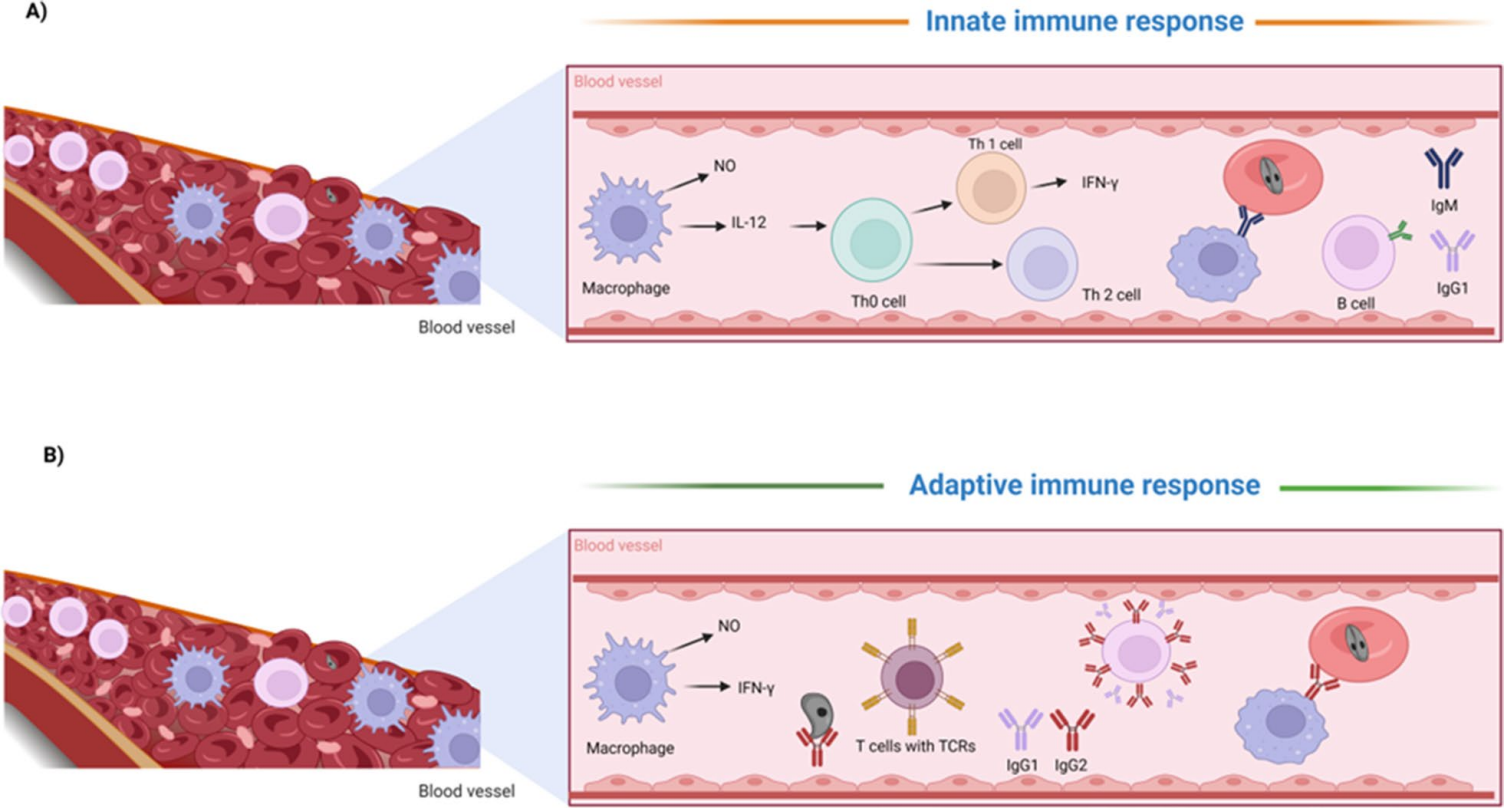
Scheme of immune responses in bovines infected with *Babesia* parasites. **A** Schematic of innate immunity. Innate immune responses in young calves are characterized by the swift activation of macrophages and the abundant release of interferon-γ (IFN-γ) and nitric oxide (NO). Young, naive calves have a natural resistance to *Babesia* infection and typically survive exposure to *Babesia*-infected ticks in endemic areas. In contrast, adult animals are more susceptible to *Babesia* infection, which often results in acute and fatal babesiosis. However, if animals survive the acute infection, they may develop chronic babesiosis and produce life-long protective immune responses. Additionally, the innate immune response is more pronounced in young animals compared to adult animals. **B** Schematic of adaptive immunity in persistently infected or vaccinated animals with live attenuated vaccine. Macrophages and protective neutralizing antibodies are deemed critical for controlling parasitemia in these animals

The induction of strong innate immune responses is necessary for immunity to *Babesia* parasites in young animals, while the development of effective adaptive immune responses is necessary for adult animals (Fig. 4). Studies have indicated that animals that have been successfully immunized with a subunit or live attenuated *B. bovis* or are persistently infected and have survived the acute stage of infection may rely on CD4^+^ T cells that are specific to the antigen and produce interferon gamma (IFN-γ) [18, 28, 29]. IFN-γ plays a crucial role in the immune response, as it can activate macrophages, is necessary for the elimination of parasites, and enhances the production of the neutralizing immunoglobulin (Ig)G2 antibody [30, 31]. Moreover, IFN-γ initiates and improves the adaptive immune response through IgG2 production by B cells, which, when combined with IgG1, protected cattle against homologous challenge [32]. Although protective neutralizing antibodies have been observed in live attenuated vaccines and persistently infected animals, the specific antigens that are targeted by these antibodies are still unknown. Moreover, the mechanism of T-cell activation and the role of distinct T-cell populations (such as γδ-T cells) in facilitating a successful adaptive immune response in vaccinated or persistently infected adult cattle requires further exploration.

## Discussion

The cattle’s immune response to *B. bovis* infection plays a vital role in the outcome of the disease, influencing the severity of clinical signs, the level of parasitemia, and the development of immunity. In this systematic review of 13 studies, which included three vaccine strategies (subunit, live attenuated, and viral vector), we found evidence of promising vaccine candidates that could be further developed to improve efficacy and temporal immunity against *B. bovis*.

Our current knowledge and understanding of the immune mechanisms underlying protection against bovine babesiosis remains inadequate due to a range of practical, ethical, and economic constraints associated with conducting bovine experiments and the lack of dependable small animal models for *B. bovis* studies. The data from our systematic review suggests that this knowledge gap impairs the development of effective vaccines since the absence of practical and reliable experimental systems limits the investigation of the underlying protective mechanisms. Despite significant advancements in comprehending the immune responses to *B. bovis* infections, this challenge persists. The ability to survive the acute stages of infection is dependent on age and robust innate immune responses, which must be followed by effective stimulation of immune mechanisms that lead to the production of antibodies. These antibodies play a critical role in controlling infection in vaccinated and persistently infected animals.

There is also an age-related immunity to primary infection with *B. bovis.* Upon initial infection with *B. bovis*, young calves (less than 6 months) are generally immune to developing serious disease as seen in vulnerable adults [31]. Initially, this phenomenon was thought to be due to passive immunity from the protective antibodies in the colostrum [33]. However, subsequent studies found that the immune response of young calves to *B. bovis* infection includes the early activation of IFN-γ and interleukin (IL)-12 and the existence of inducible nitric oxide synthase (iNOS) messenger RNA (mRNA) expression in the spleen [34]. In contrast, iNOS was not induced, and IFN-γ and IL-12 were activated later in *B. bovis* infection in adult cattle. Another possible explanation for increased resistance in calves is the presence of a high proportion of γδ-T cells, which encompasses up to 70% circulating T cells in calves and 30% in adult cattle [35] or reduced pro-inflammatory cytokines in response to infection which may aid in pathogenesis [36]. In addition, the spleen is an important organ in controlling the infection as indicated by splenomegaly during acute babesiosis, and elevated levels of parasitemia in splenectomized animals [34]. The effective activation of innate immune mechanisms in young animals can result in the development of a protective adaptive immune response in adult animals, which prevents the establishment of persistent disease or death due to the detrimental effects of uncontrolled acute infection. A deeper understanding of the mechanisms that confer resistance against acute *B. bovis* infection in young and naïve animals (i.e., innate immunity), as well as those necessary to control parasitemia to persistently low levels in adult cattle that have survived the infection (i.e., adaptive immunity), is crucial for the development of vaccination strategies. Further research is essential to address this important research gap.

### Progress and challenges in *B. bovis* vaccine development

As babesiosis significantly affects the animal and livestock industry, more effective control of this parasite would decrease the burden of disease [13]. The primary obstacle in controlling babesiosis is the lack of effective vaccines and the development of anti-babesial drug resistance [8, 13].

Common strategies for controlling babesiosis include tick management, anti-babesial drugs, and administration of live attenuated vaccines [26]. In Australia, live attenuated vaccines are generated in splenectomized calves, while in South America, in vitro culture of the *Babesia* parasites has been successfully utilized and has paved the way for vaccine development. Currently, available live attenuated vaccines that contain viable *Babesia*-infected RBCs have several limitations, such as the transmission risk of contaminating blood-borne pathogens and the risk of reversion of the *Babesia* parasite to a virulent phenotype [26]. Additionally, it is also a problem to keep the donor cattle *Babesia* spp.-free when vaccine preparations or production are carried out in tick-endemic countries [9]. Lastly, the vaccine needs a longer shelf-life and a cold chain to retain its efficiency, which is a challenge in tropical regions [18].

*Babesia* parasites produced in vitro have been a novel strategy for developing a vaccine against *B. bovis* [9]. Vaccines produced by in vitro methods are less likely to transmit pathogens, and at the same time, they allow more controlled and standardized conditions [37]. However, the critical limitation of this method is that it needs a constant supply of serum and erythrocytes from donor animals as well as sufficient laboratory equipment and trained staff [9]. It was found that vaccines based on live *B. bovis* did not result in an immune response that could eliminate the parasite, but rather generated a disease-resistant carrier that could serve as a reservoir for tick transmission [38]. Despite the progress made in vaccine development against *B. bovis*, several gaps in knowledge remain. A significant gap is the lack of understanding of cattle's immune response to *B. bovis* infection. The role of T cells and cytokines in protective immunity is not well understood, and further research is needed to elucidate the mechanisms of immune protection. As highlighted above, immune responses to *Babesia* involve innate and adaptive immune systems.

In comparison with mammals, arthropods lack the capacity to develop adaptive immune responses, and their immune system is less complex. Moreover, *Babesia* parasites have co-evolved with ticks and with their hosts by developing the ability to undergo biological amplification and sexual reproduction without affecting and stimulating the tick immune system. However, studies focusing on the modulation of *Babesia* proteins in tick immunity are lacking and some research with other invertebrates has provided insights into the immune system of ticks [39]. Altogether, it is tempting to speculate that *Babesia* proteins that regulate the tick’s immune system to initiate infection could be ideal candidates for transmission-blocking vaccines, but these antigens are yet to be identified. Further, more research is needed on host antigen presentation to stimulate CD4^+^ T cells and provide helper B cells. Better knowledge and understanding of the innate immune responses in naïve and young animals infected with *B. bovis*, antibody responses in adult animals with high levels of parasitemia, and tick immune responses to *Babesia* parasites are vital to developing and designing strategies to induce protective immunity, and thus further research is warranted to close this critical gap of knowledge. Furthermore, it is important to note that the limited understanding of the genetic diversity of *B. bovis* strains is another knowledge gap. The efficacy of vaccines could be impacted by the genetic diversity of the parasite, underscoring the need for further research to characterize the genetic diversity of *B. bovis* strains worldwide. Therefore, closing these critical knowledge gaps is essential to design effective strategies for inducing protective immunity against *B. bovis* infection.

The safety and efficacy of vaccines in field conditions are also critical gaps in knowledge. Vaccine efficacy studies often rely on experimental challenge studies in controlled environments, which may not reflect the real-world conditions of cattle production. Further studies are needed to evaluate the safety and efficacy of vaccines under field conditions and in different cattle populations.

### Modern molecular toolkit and future directions

The prevention and management of bovine babesiosis present significant biological challenges and research gaps that need to be addressed for the development of effective control methods. However, the continuous influx of breakthroughs and technological advancements in the field of both bovine babesiosis and closely related apicomplexans supports the successful development of innovative methods aimed at more efficient control of bovine babesiosis. For instance, the sequencing of relevant *Babesia* spp. genomes, beginning with the publication of the first complete *B. bovis* genome in 2007, has resulted in important advances in our understanding of parasite biology [40]. These advancements have been facilitated by the development of transfection systems for gene modification and functional analysis, accelerating vaccine candidate discovery [13]. As a result of genomic advancements, critical genes and gene families involved in host immune evasion and sexual stage development, such as the large and antigenically variable *ves1* gene family [41], 6-Cys [42], CCp1-3 [43], CPW-WPC [44], and HAP2 [45], have been completely identified [7]. Additionally, conserved master regulatory genes such as AP2 [46] have been identified and found to be responsible for transcriptional control of genes involved in parasite stage transitions, similar to what has been observed in *Plasmodium* and *Theileria* parasites [46, 47].

The use of “omic” techniques, such as transcriptomics, enabled the comparison of virulent and attenuated *Babesia* spp. strains to identify virulence factors and attenuation markers, a critical research gap in developing effective vaccines [13]. The implementation of these techniques holds promise in identifying the peptides presented by major histocompatibility complex (MHC) class II molecules to CD4^+^ T cells and genes that are differentially expressed during the different stages of the parasite's life cycle, thereby facilitating the discovery of novel and promising vaccine targets.

Altogether, the information and advanced genetic manipulation techniques will play a crucial role in creating innovative vaccines that could target the different stages of the *Babesia* parasite's life cycle. This approach may be critical for effectively managing the disease in the future.

## Conclusions

The biology of *Babesia* parasites has been a subject of intense research interest over the years, owing to their ability to cause babesiosis in humans and animals. The advancement of research in this area has been heavily influenced by the advances in molecular and cellular biology, immunology, computational sciences, and vaccinology. In this systematic review, we provided an overview of the current state of knowledge regarding *B. bovis* vaccine development studies and highlighted the areas that require further investigation.

A complete understanding of the biology of *Babesia* parasites is critical for the development of effective strategies for controlling bovine babesiosis. This includes a better and more detailed characterization of the different distinct phases in the parasite’s life cycle, and the interactions between *Babesia* parasites and their host. Recent advances in genomic and proteomic technologies have provided a wealth of information on the biology of *Babesia* parasites, including their virulence factors, antigenic variation, and host–parasite interactions. However, several challenges need to be addressed. In practical terms, research in the biology of *Babesia* parasites will require multidisciplinary approaches, including the integration of molecular and cellular biology, immunology, computational sciences, and vaccinology. The availability of these tools provides an appropriately equipped toolbox for guiding researchers on a successful journey towards bovine babesiosis control. Overall, the advancement of research in the biology of *Babesia* parasites is critical for the development of effective strategies for controlling bovine babesiosis. The multidisciplinary approaches and the availability of advanced technologies provide an optimistic outlook for future research in this area. However, the development of an effective vaccine remains a critical challenge that warrants further attention.

### Supplementary Information

**Additional file 1: **Keywords and search strategies for each database

## Data Availability

Not applicable.
