# Supplementary material for: Exploring the landscape of Babesia bovis vaccines: progress, challenges, and opportunities
Source: Parasit Vectors. 2023 Aug 10;16:274. doi: 10.1186/s13071-023-05885-z (PMC10413621; doi:10.1186/s13071-023-05885-z)
Supplement: Supplementary file 1 — Additional file 1: Keywords and search strategies for each database [file 13071_2023_5885_MOESM1_ESM.docx]

**Supplementary File 1: Keywords and search strategies for each database**

**PubMed (Title and abstract)**

(((((((((((cattle[Title/Abstract]) OR (bovine[Title/Abstract])) AND (vaccine[Title/Abstract])) OR (vaccines[Title/Abstract])) OR (vaccination[Title/Abstract])) OR (live attenuated[Title/Abstract])) OR (subunit[Title/Abstract])) OR (recombinant[Title/Abstract])) OR (inactivated[Title/Abstract])) AND (babesia bovis[Title/Abstract])) OR (babesiosis[Title/Abstract])) AND (immune response[Title/Abstract])

**Web of Science Core Collection (Topic)**

(Topic) OR bovine (Topic) AND vaccine (Topic) OR vaccines (Topic) OR vaccination (Topic) OR live attenuated (Topic) OR subunit (Topic) OR recombinant (Topic) OR inactivated (Topic) AND immune response (Topic) AND babesia bovis (Topic) OR babesiosis (Topic)

**Scopus (Title, abstract and keyword)**

(TITLE-ABS-KEY (cattle) OR TITLE-ABS-KEY ( bovine ) AND TITLE-ABS-KEY ( vaccine ) OR TITLE-ABS-KEY ( vaccines ) OR TITLE-ABS-KEY ( vaccination ) OR TITLE-ABS-KEY ( live AND attenuated ) OR TITLE-ABS-KEY ( subunit ) OR TITLE-ABS-KEY ( recombinant ) OR TITLE-ABS-KEY ( inactivated ) AND TITLE-ABS-KEY ( babesiosis ) OR TITLE-ABS-KEY ( babesia AND bovis ) AND TITLE-ABS-KEY ( immune AND response ) )

**Embase (Title, abstract and keyword)**

(((bovine:ti,ab,kw OR cattle:ti,ab,kw) AND vaccine:ti,ab,kw OR vaccines:ti,ab,kw OR vaccination:ti,ab,kw OR 'live attenuated':ti,ab,kw OR subunit:ti,ab,kw OR recombinant:ti,ab,kw OR inactivated:ti,ab,kw) AND babesiosis:ti,ab,kw OR 'babesia bovis':ti,ab,kw) AND 'immune response':ti,ab,kw
